# The Effect of Montmorillonites on the Physicochemical Properties of Potato Starch Films Plasticized with Deep Eutectic Solvent

**DOI:** 10.3390/ijms232416008

**Published:** 2022-12-16

**Authors:** Dorota Skowrońska, Katarzyna Wilpiszewska

**Affiliations:** Department of Chemical Organic Technology and Polymeric Materials, Faculty of Chemical Technology and Engineering, West Pomeranian University of Technology in Szczecin, 70-322 Szczecin, Poland

**Keywords:** starch, plasticization, deep eutectic solvent, montmorillonite

## Abstract

In the paper, the method of obtaining the potato starch nanocomposites plasticized with a deep eutectic solvent is described. The deep eutectic solvent based on choline chloride and malic acid (CM, molar ratio 1:1) was used as the plasticizer. The effect of the sodium and calcium montmorillonite (MMTNa, MMTCa respectively) addition on the properties of potato starch films was investigated. The thermal, mechanical, and barrier properties were determined. Moreover, a moisture absorption test was performed. The starch gelatinization temperature increased in the presence of montmorillonite. The values of glass transition determined by DMTA depended on the nanofiller type. For the systems containing MMTCa, they generally decreased with its content (although still lower than reference samples). The obtained nanocomposites showed improved mechanical and barrier properties. The highest values of tensile strength and Young’s modulus were noted for the system containing 1% MMTNa. The XRD revealed that only the films with MMTNa exhibited intercalation. The homogeneity of the samples decreased with increasing nanofiller concentration. This was probably due to the occurrence of choline chloride-montmorillonite interactions, which were more favored than clay-starch interactions.

## 1. Introduction

Plastic pollution is one of the most frequently raised environmental problems. Numerous reports have identified the main sources of pollution. The need to increase the proportion of biodegradable plastics was pointed out [1]. Next to cellulose, starch is one of the most abundant biopolymers in nature [2]. There are many starch sources, and it is biodegradable and easy to isolate. Starch consists of two types of macromolecules: linear amylose—anhydroglucose linked by α-(1,4)-glycosidic bonds, and branched amylopectin—with an additional α-(1,6)-glycosidic. Each repetitive unit contains three hydroxyl groups, one primary and two secondary, thus numerous hydrogen bonds between the starch chains could be formed. Due to the extensive hydrogen bonding network, the melting point of starch is higher than its degradation temperature. It could be lowered by introducing low molecular weight additives (plasticizers) capable of replacing polymer-polymer interactions in favor of polymer-plasticizer ones. In the presence of a plasticizer, at elevated temperature, the granular structure of starch is destroyed. This process is called gelatinization and results in a material (thermoplastic starch) exhibiting lower crystallinity, as well as melting and glass transition temperatures [3]. The most commonly applied starch plasticizers are polyols (e.g., glycerol), nitrogen compounds (e.g., urea), and sugars (e.g., glucose, fructose) [4]. However, recently the use of deep eutectic solvents for this purpose has been reported [5].

Deep eutectic solvents are eutectic mixtures of two or more components, and their melting temperature is significantly lower (deep) compared to the melting temperatures of the components. This is due to the interactions between the components of the mixture. Currently, five groups of deep eutectic liquids could be distinguished: I: mixtures of quaternary ammonium salts and metal chlorides; II: mixtures of quaternary ammonium salts and metal chloride hydrates; III: mixtures of quaternary ammonium salts and hydrogen bond donors (e.g., polyols, amines, carboxylic acids); IV: mixtures of metal chloride hydrates and hydrogen bond donors; and V: mixtures of non-ionic hydrogen bond acceptors and donors [6].

The drawbacks of thermoplastic starch materials, such as their low resistance to moisture or insufficient mechanical properties, could be overcome by applying nanofillers. According to the shape, a wide range of nanometric structures is known, and includes 1D nanowires and nanotubes, 2D nanosheets, and 3D powders [7,8,9,10]. Obtaining the composites from thermoplastic starch with, for example, cellulose nanocrystals, nanofibers, and montmorillonites (natural and modified), were reported [11]. The starch nanocomposites could be prepared by extrusion [12,13,14] or casting [15,16,17,18,19,20,21], and glycerol was the most commonly used for plasticization. Applying the polyols was also described [22,23,24,25]. However, the research on preparing nanocomposites based on starch plasticized with deep eutectic solvents (choline chloride:glycerol molar ratio 1:2 and choline dihydrogencitrate:glycerol:urea molar ratio 1:2:2) has been reported only in one work so far [26]. The presence of montmorillonite (MMT) resulted in the improvement of the strength and barrier properties when the starch chains could migrate between the montmorillonite layers (intercalation or even exfoliation occurred). It seemed that a proper dispersion of the filler was essential for the final starch-based product. However, the presence of a plasticizer in such a composite could lead to some issues. Due to its small particle size, the plasticizer could penetrate between the montmorillonite layers and thus lower the possibility of interactions between starch and filler. Therefore, the order in which starch, clay, and plasticizer are added to the polysaccharide system is essential for homogeneous filler dispersion. It has been recommended to gelatinize the starch in the presence of clay, followed by the addition of the plasticizer [15].

In this paper, the method of obtaining the potato starch nanocomposites plasticized with a deep eutectic solvent was described. The deep eutectic solvent based on choline chloride and malic acid (CM, molar ratio 1:1) was used. The effect of montmorillonite type (sodium, or calcium), and content on the physicochemical properties (thermal, mechanical, moisture vulnerability) of starch films was determined. Due to the significant differences in suspension viscosity between the montmorillonites, different MMTCa and MMTNa contents were applied. The films based on plasticized starch with MMT were prepared, and the systems containing 1% of the nanofiller were used to compare the effect between the montmorillonites. This kind of material could be considered for applications such as food packaging, edible coatings or in agriculture.

## 2. Results and Discussion

### 2.1. Modulated Differential Scanning Calorimetry (MDSC)

**Figure 1:**
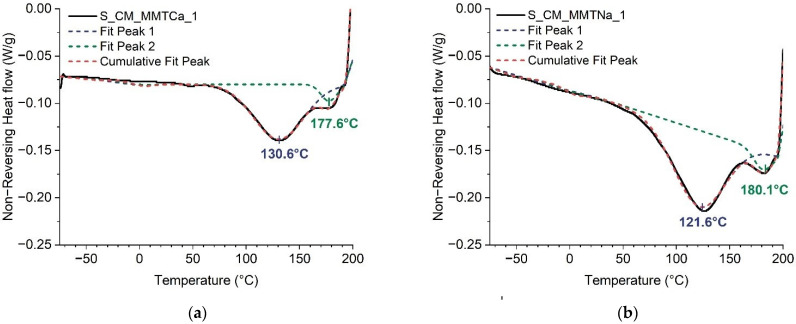
MDSC thermograms of non-reversing transition with curve deconvolution of starch films with 1% of (**a**) calcium montmorillonite, (**b**) sodium montmorillonite.

The effect of the nanofiller type and content on the thermal properties of starch systems plasticized with a deep eutectic solvent was tested. Due to the possibility of overlapping the phase transformations, it was decided to use modulated differential scanning calorimetry, as the separation of the reversible and non-reversible transformations was available. In Table 1 the temperature values of non-reversible transition were collected (sample abbreviations were created as follows: S_CM_ filler type_filler content). In the thermogram of the reference sample, two endothermal peaks were noted. The first one (T_gel_) corresponded to starch gelatinization and the second one, (T_r_) was probably the result of the reaction between starch and the plasticizer (esterification between starch hydroxyl groups with carboxylic groups of malic acid). In the case of the starch films containing MMT the signals overlapped, therefore the deconvolution of the thermograms was performed (Figure 1). The addition of montmorillonite resulted in a significant increase in gelatinization temperature (ca. 131 °C) when compared to the reference (ca. 78 °C), which could indicate the interaction between silicate-plasticizer or/and silicate-starch. The slightly lower gelatinization temperature increase was noted for sodium montmorillonite. The difference in melting temperatures may be due to differences in the interaction of montmorillonites with the plasticizer. It is probable that some part of the plasticizer interacted with the nanofiller (absorbed) and the reduced amount of plasticizer was available for starch plasticization. It seemed that the plasticizer exhibited higher affinity to calcium montmorillonite than to sodium montmorillonite. 

With increasing montmorillonite content, determining the values of transition temperatures was even more difficult due to the signals overlapping. It seemed that they were moving towards higher values, but it was difficult to make a clear statement.

### 2.2. Dynamic Mechanical Thermal Analysis (DMTA)

**Figure 2:**
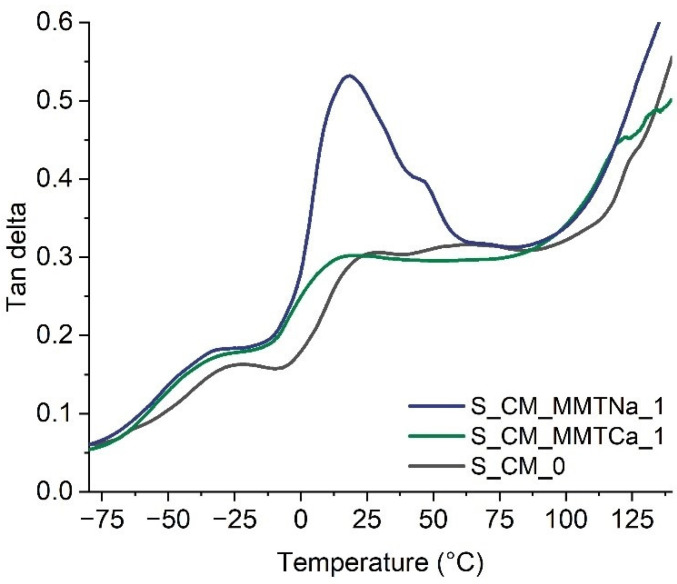
DMTA thermograms of starch samples.

DMTA allows the determination of the phase transition temperatures, even when they could not be noted on DSC thermograms. Moreover, the compatibility between the system components could be evaluated. In DMTA thermograms of starch-based films, two transitions could be distinguished (the values are collected in Table 2). The one at the lower temperature, ca. −30 °C, referred to the plasticizer-rich phase and the glass transition of the plasticizer (T_β_). The higher temperature transition corresponded to the starch glass transition (T_α_). In the case of S_CM_MMTCa systems, the T_α_ generally increased with nanofiller content, but only the sample with the highest content of calcium montmorillonite (S_CM_MMTCa_7) exceeded the T_α_ of the reference sample. It could suggest a more plasticizer-like activity of the nanofiller. The MMTCa probably formed the aggregates that worked as a lubricant, facilitating the movement of polysaccharide chains (according to one of the theories of plasticization) [27]. However, for the highest MMTCa content the T_α_ value increased, which could be the result of a crosslinking reaction between malic acid (a component of plasticizer) [26]. For the films with MMTNa, no apparent trend between filler content and T_α_ was observed. The films containing MMTNa up to 1.5% exhibited the T_g_ similar to or slightly higher than the reference. However, the highest nanofiller content resulted in a reduction of the value of this parameter. It could suggest some interactions with the plasticizer. In Figure 2 the thermograms of reference film (S_CM_0) and those containing 1 wt% of montmorillonite (S_CM_MMTCa_1 and S_CM_MMTNa_1) are shown. For S_CM_MMTNa_1, the peak is much more intense, which could indicate better dispersion of the nanofiller in the films [23]. Thus, the XRD measurements were necessary to evaluate the degree of MMT dispersion in the starch matrix.

### 2.3. Fourier Transform Infrared Spectroscopy (FTIR-ATR)

**Figure 3:**
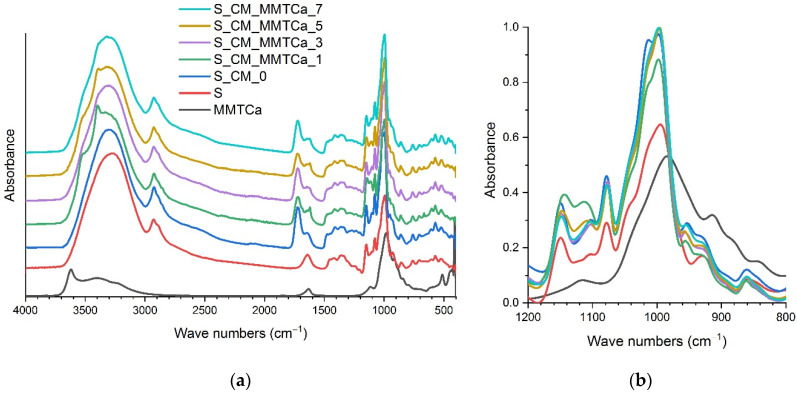
FTIR–ATR spectra of calcium montmorillonite, starch and films with various nanofiller contents: (**a**) full spectrum, (**b**) range 1200–800 cm^−1^.

**Figure 4:**
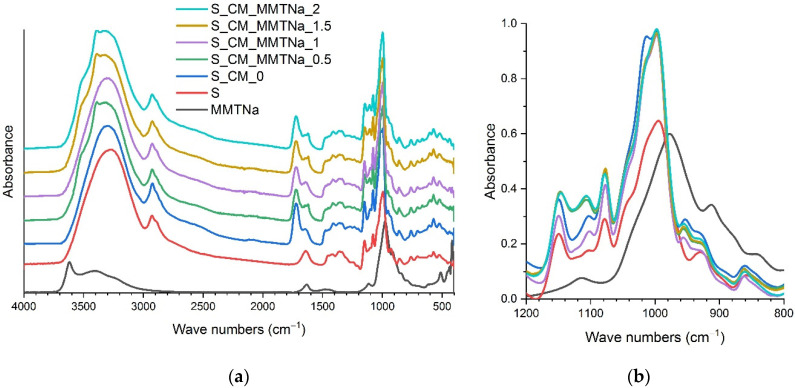
FTIR–ATR spectra of sodium montmorillonite, starch and films with various nanofiller contents: (**a**) full spectrum, (**b**) range 1200–800 cm^−1^.

FTIR spectra of starch films plasticized with deep eutectic solvent based on choline chloride and malic acid with various calcium and sodium montmorillonite content were shown in Figure 3 and Figure 4, respectively. The typical starch absorption bands were identified, e.g., a wide band in the range of 3000–3500 cm^−1^ assigned to the hydroxyl group, at 2900 cm^−1^ to the C-H bond, at 1650 cm^−1^ from absorbed water, and in the range of 900–1200 cm^−1^ assigned to the C-O stretching band. Importantly, a new band around 1013 cm^−1^ was observed, indicating an increase of the amorphous phase content in the starch-based films (Figure 3b and Figure 4b) [28]. By comparing the spectrum of native starch and starch films plasticized with a mixture of choline chloride and malic acid, the new bands around ca. 1720 cm^−1^ assigned to carbonyl groups, 1475 cm^−1^ C-H groups, and 950 cm^−1^ C-OH could be noticed (coming from the plasticizer). Moreover, the band at 1650 cm^−1^ shifted towards lower values (ca. 1620 cm^−1^). The spectra were taken at three different locations. However, no clear correlation was observed between the additive content and the observed differences, which were presented in previous works [15,22,26]. These must have been due to the higher amount of montmorillonite present locally. A decrease in band intensity ca. 1720 cm^−1^ was observed for all materials containing montmorillonite, which could be the result of the interactions between the plasticizer and montmorillonite [26,29].

### 2.4. X-ray Diffraction (XRD)

**Figure 5:**
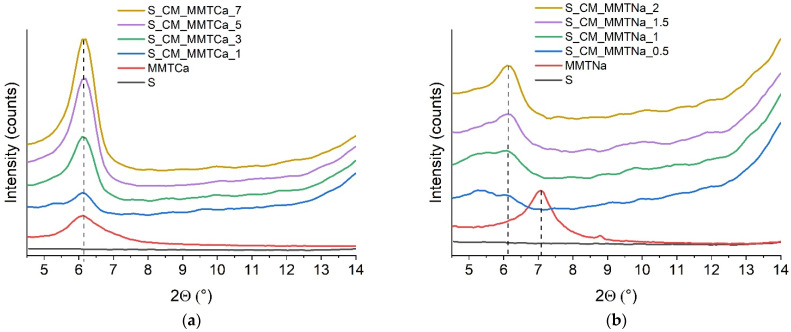
XRD diffractograms of neat montmorillonite and starch films with various (**a**) MMTCa, and (**b**) MMTNa contents.

In Figure 5, the XRD patterns of the reference sample, montmorillonites, and starch films with various montmorillonite content were presented. The diffraction signals for neat nanofiller were at ca. 6° and 7° for MMTCa and MMTNa, respectively. For the films containing MMTCa, no shift in peak position was observed. Additionally, with increasing the nanofiller content, the intensity of the signal increased. It directly indicated poor dispersion of the nanofiller in the polysaccharide matrix. These results correlated with DMTA thermograms. In the case of starch films with MMTNa, the signals were shifted to lower values of 2Θ, indicating the intercalation of montmorillonite. Moreover, the d-spacing calculated for the samples with 2% MMTNa was 1.85 nm (for neat MMTNa 0.63 nm) [12]. For materials containing lower filler content (0.5 and 1%), the signals flattened, as it could be noticed for exfoliation; however, it was probably the result of the low MMTNa amount.

### 2.5. Moisture Absorption

**Figure 6:**
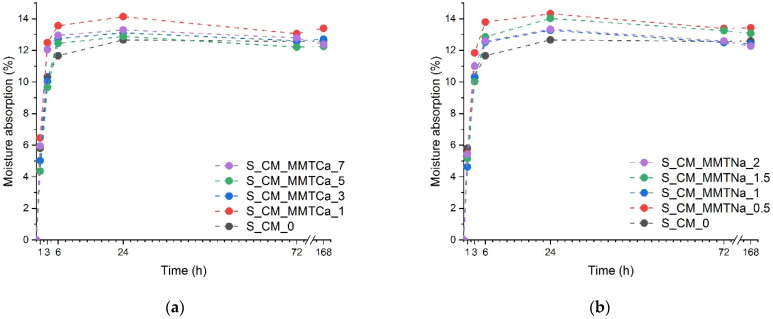
Water absorption of starch films containing (**a**) calcium montmorillonite, and (**b**) sodium montmorillonite.

In Figure 6 the moisture absorption results are presented. Generally, the addition of montmorillonite resulted in increased moisture absorption of the starch films when compared with the reference material. Interestingly, the films with the lowest amount of nanofiller exhibited the highest moisture absorption, whereas the samples with a higher montmorillonite content reached intermediate values. It was the opposite of other works [14,15,16,17] in which glycerol was used as a plasticizer, and moisture absorption decreased with the amount of nanofiller. The moisture uptake depends on both the hydrophilicity and morphology of the tested material. Unmodified montmorillonites are more strongly hydrophilic. In the tested system, the filler probably interacted with the plasticizer, thus the MMT platelets could not be evenly spread in the starch matrix. It is known that well-dispersed MMT hindered water penetration [15]. The inhomogeneous filler arrangement resulted in the opposite, i.e., an increase in moisture absorption. Larger agglomerates absorbed water on the surface, but the penetration inside was impeded. Hence, with filler content, the moisture absorption decreased.

### 2.6. Water Vapor Transmission Rate (WVTR)

**Figure 7:**
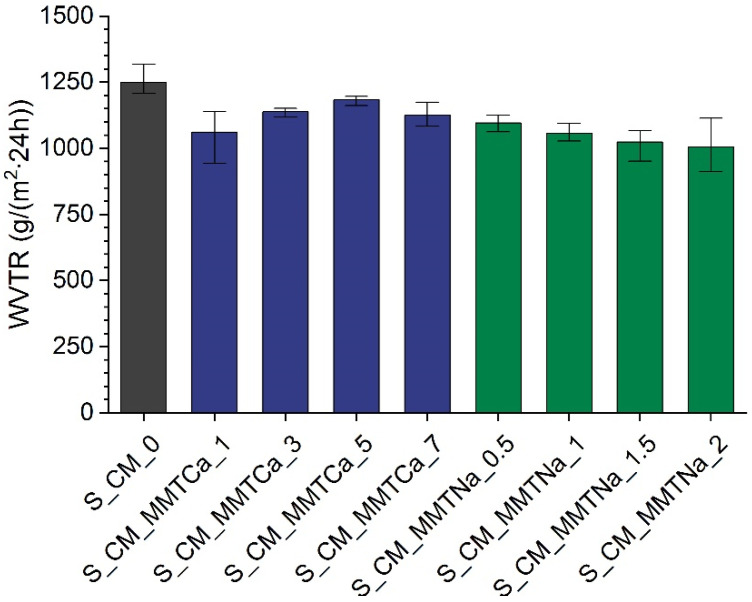
Water vapor transmission rate of starch films with various calcium and sodium montmorillonite contents.

Generally, introducing montmorillonite resulted in a decrease of water vapor transmission rate (Figure 7). For films with sodium montmorillonite, it decreased with a nanofiller content, and it was similar to glycerol-plasticized starch systems containing montmorillonite [13]. However, the films with MMTCa up to 5 wt% showed the opposite trend, i.e., WVTR increased with increasing nanofiller content. Additionally, the system with the highest MMTCa content exhibited reduced WVTR. The probable reason could be the agglomeration of nanofiller, so the penetration of water molecules was hindered. The results correlate with those obtained for the moisture absorption measurements.

### 2.7. Mechanical Properties Test

**Figure 8:**
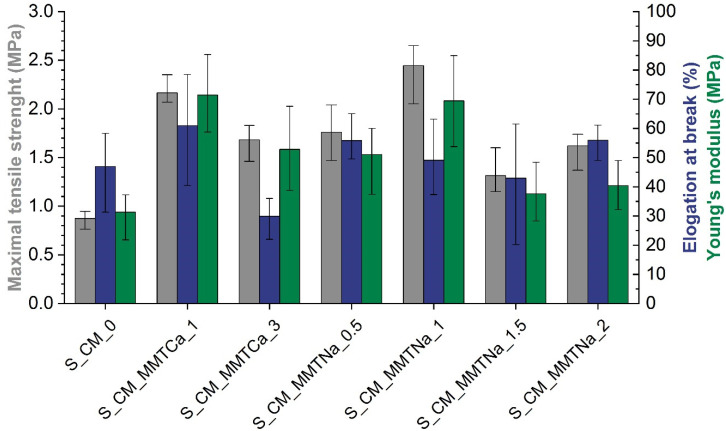
Mechanical properties of starch films with calcium (MMTCa) or sodium (MMTNa) montmorillonite (gray—maximal tensile strength (MPa), blue—elongation at break (%), green—Young’s modulus (MPa)).

The results of the mechanical tests are presented in Figure 8. With the nanofiller content’s increase, the tensile strength and Young’s modulus of starch materials were improved, but the elongation decreased [14,17]. In the case of calcium montmorillonite materials, the systems containing 1% filler exhibited the highest tensile strength and Young’s modulus, i.e., 2.17 MPa and 71 MPa, respectively. With an increase in the filler content, the homogeneity of the samples was limited, and as a consequence, the values of the mechanical parameters decreased. In the case of sodium montmorillonite, the systems with 1 wt% also exhibited the highest tensile strength and Young’s modulus values, i.e., 2.45 MPa and 69 MPa, respectively. It was reported that the MMT addition improved the strength and Young’s modulus but reduced flexibility [14,16,22], and the better the dispersion, the better the material properties [13]. The systems containing 1% MMT exhibited the highest mechanical improvement, which may also indicate better dispersion of the filler at this concentration. Moreover, the intercalation of silicate layers does not necessarily lead to a reduction in elongation values [15], which is evident in samples containing 1% MMT. 

### 2.8. Laser Scanning Microscopy (LSM)

**Figure 9:**
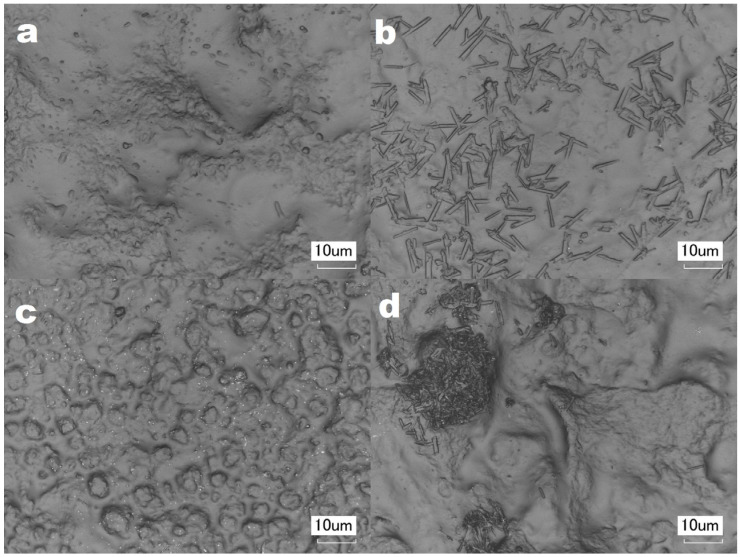
Microphotographs (magnification of 3000×) of starch films plasticized with deep eutectic solvent: with the addition of 1% (**a**) sodium (S_CM_MMTNa_1) or (**b**) calcium (S_CM_MMTCa_1) montmorillonite, (**c**) without filler (S_CM_0), (**d**) with addition of 7% of calcium montmorillonite (S_CM_MMTCa_7).

The sample morphology was analyzed using laser scanning microscopy. The microphotographs of starch nanocomposites are presented in Figure 9. On the surface of the MMTCa films, some elongated particles can be observed (Figure 9b). This is probably because of the interactions between MMTCa and the deep eutectic solvent’s components. They could be absorbed by the MMTCa aggregates and then crystallize. The particles were evenly distributed on the film surface. Interestingly, the particles were not observed on the starch films containing MMTNa (Figure 9a). The nanofiller content was the same, thus the interaction with MMTCa was more intense than with MMTNa. These observations correlated with the MDSC results.

Additionally, the microphotographs of the films with higher montmorillonite content (Figure 9d) revealed that in the case of S_CM_MMTCa_7 the particles formed bigger aggregates. This indirectly indicated filler aggregation. In the case of MMTNa, (S_CM_MMTNa_2) the single particles were noted. Thus, it was concluded that with increasing MMTNa content the degree of dispersion reduced. However, MMTNa could be better dispersed in the starch matrix than MMTCa, which correlated with XRD results.

## 3. Materials and Methods

### 3.1. Materials

The deep eutectic solvent components were choline chloride (TCI Chemicals, Tokyo, Japan) and malic acid (Aldrich Chemistry, China). Starch was purchased from Zetpezet (Piła, Poland). Montmorillonites (Ca and Na) were obtained from ZGM (Zębiec, Poland).

#### 3.1.1. Deep Eutectic Solvent Preparation

Choline chloride and malic acid (molar ratio 1:1) were placed into a glass vessel and sealed. The mixture was mixed for ca. 0.5 h by magnetic stirrer in a water bath at 80 °C to obtain a homogenous liquid. The eutectic liquid was marked as “CM”.

#### 3.1.2. Films Preparation

Depending on the montmorillonite type, various nanofiller amounts were introduced into the starch matrix, i.e., for calcium montmorillonite: 1, 3, 5, and 7% (per dry weight of starch), and for sodium montmorillonite 0.5, 1, 1.5, and 2%. The reason was the high viscosity of the MMTNa aqueous suspension. First, the filler aqueous suspension was mixed (500 rpm) for 30 min and subsequently sonified for 10 min (Hielscher UP200, 200 W, 24 Hz, amplitude 60%, cycle 0.5). Starch (S) was then added, and the system was stirred for 30 min at 90 °C in a water bath. Next, the deep eutectic solvent (CM) (30% calculated per dry weight of starch) was added, and the system was stirred for another 10 min (without heating). The starch mixture was cast on a Petri dish and dried at 50 °C. The films were placed in a climate chamber for at least 24 h before testing.

### 3.2. Methods

#### 3.2.1. Modulated Differential Scanning Calorimetry (MDSC)

The thermal properties of the films were determined by modulated differential scanning calorimetry. The mixture of starch with the plasticizer and nanofiller (ca. 10 mg) was placed in the aluminum hermetic pan. The tests were performed on DSC250 (TA Instruments) in the temperature range from −80 °C to 250 °C, and a heating rate of 10 °C/min (1 cycle).

#### 3.2.2. Dynamic Mechanical Thermal Analysis (DMTA)

Dynamic mechanical thermal analysis tests were performed using DMA Q800 from TA instruments. The measurements were performed using film tension mode, at a frequency of 1 Hz, a heating rate of 3 °C/min, and a temperature range from −90 to 140 °C. The transition temperatures (tan δ, storage, and loss modulus) were analyzed with TA Universal Analysis software.

#### 3.2.3. Fourier Transform Infrared Spectroscopy with Attenuated Total Reflection (ATR-FTIR)

The spectra of starch films were obtained using a Nexus FTIR Spectrometer (Thermo Nicolet corp., Waltham, MA, USA) equipped with attenuated total reflectance. Each sample was scanned 32 times in a wave number range from 4000 to 400 cm^−2^. The spectra were analyzed with OMNIC software.

#### 3.2.4. X-ray Diffraction (XRD)

Diffraction patterns were performed on an Empyrean X-ray diffractometer from PANalytical (Almelo, Holland) with Cu K_α_ radiation. The scanning range of the diffraction angle 2θ was from 4.5 to 70°. The d-spacing was calculated from Bragg’s equation, where λ = 0.154 nm.

#### 3.2.5. Moisture Absorption

After drying (24 h in 60 °C), starch films (20 × 10 mm) were weighed (m_0_) and placed in the climate chamber (RH 50%, 30 °C). The samples were weighed (m_w_) after 1, 3, 6, 24, 72 and 168 h. Moisture absorption was calculated from the following equation:

(1) $$\mathrm{moisture}\;\mathrm{absorption}=\frac{\left(m_{w}\;{-\;m}_{0}\right)}{m_{0}}\;\times \;100\%$$

#### 3.2.6. Water Vapor Transmission Rate (WVTR)

The gravimetric dish method was used to measure the water vapor transmission rate (according to ISO 2528 standard). The samples were conditioned in a climate chamber for 24 h before the test. Silica gel was used as a desiccant. The test used an Elcometer 5100 Payne Permeability Cup (area 10 cm^2^). The samples were weighed before and 24 h after placing them in a climate chamber (RH 70%, 23 °C). The WVTR was calculated from the following equation:

(2) $$\mathrm{WVTR}=\frac{{240\;\times \;m}_{1}}{S}$$

where m_1_ is the rate of mass increase in time determined from the graph (mg/h), and S is an area of the tested surface (cm^2^).

#### 3.2.7. Mechanical Properties

Tensile strength measurements were performed using an Instron 5982 in accordance with the standard PN-EN ISO 527-3. The initial gap separation was 50 mm and the cross-head speed was 10 mm/min (with load cell 1 kN). At least six replicants of the starch film stripes (5 × 100 mm, ca. 0.3 mm thick) were tested. The mechanical parameters (maximal tensile strength, elongation at break, and Young’s modulus) were determined with Bluehill 3 software (Instron, Opole, Poland).

#### 3.2.8. Laser Scanning Microscopy (LSM)

The microphotographs of starch samples were performed by a laser scanning microscope model VK 9700 (Keyence, Mechelen, Belgium) equipped with a violet laser source (wavelength 408 nm) and a pinhole optical system.

## 4. Conclusions

Starch films with calcium and sodium montmorillonite were obtained using the casting method. The effect of the montmorillonite type on the starch-based composites was evaluated. The thermal properties of the nanocomposites were tested. The starch gelatinization temperature increased in the presence of MMT. The values of glass transition determined by DMTA depended on the nanofiller type. For the systems containing MMTCa, it generally decreased with its content. The films containing MMTNa up to 1.5% exhibited similar T_g_ values or slightly higher ones than the reference. The XRD measurements were applied to evaluate the degree of MMT dispersion, and only the films with MMTNa exhibited intercalation. The obtained nanocomposites showed improved mechanical and barrier properties. The highest values of tensile strength and Young’s modulus were noted for the system containing 1% MMTNa, i.e., 2.45 MPa and 69 MPa, respectively. The homogeneity of the samples decreased with increasing nanofiller concentration. The microscopic analysis revealed the formation of crystal particles on the surface of the composites containing MMTCa (they were uniformly distributed for lower nanofiller dosage). Interestingly, in the case of the films with MMTNa, the single particles could be noted only for the higher nanofiller content. The probable explanation for the differences in physicochemical properties of the nanocomposites with various nanofillers was the plasticizer-MMT interaction. Such materials can be a potential biofriendly replacement for some of the currently used non-biodegradable plastics, especially for food packaging. 

## Figures and Tables

**Table 1:** Temperature values of non-revisable transitions.

Sample Acronym	T_gel_ (°C)	T_r_ (°C)
S_CM_0	77.8	170.6
S_CM_MMTCa_1	130.6	177.6
S_CM_MMTNa_1	121.6	180.1

**Table 2:** The transition temperatures (DMTA tan δ) of starch films with montmorillonite.

Sample Acronym	T_β_ (°C)	T_α_ (°C)
S_CM_0	−29.5	20.4
S_CM_MMTCa_1	−40.6	10.6
S_CM_MMTCa_3	−35.6	16.0
S_CM_MMTCa_5	−35.6	14.3
S_CM_MMTCa_7	−31.5	23.0
S_CM_MMTNa_0.5	−32.7	21.2
S_CM_MMTNa_1	−29.6	20.0
S_CM_MMTNa_1.5	−32.1	22.9
S_CM_MMTNa_2	−27.1	17.2

## Data Availability

Not applicable.
